# Prediction model for abnormal joint line obliquity after open wedge high tibial osteotomy using knee radiographic parameters

**DOI:** 10.1186/s43019-026-00333-5

**Published:** 2026-08-04

**Authors:** Kritsada Sukha, Witoon Thremthakanpon, Burin Sutthapakti, Kriangkamol Benjawongsathien, Thanawut Hirunthanawiwat, Wiboon Wanitcharoenporn, Thisayapong Inta-Ngam, Artit Laoruengthana

**Affiliations:** 1 Department of Orthopedics, Buddhachinaraj Hospital, Phitsanulok, Thailand; 2 Phitsanulok Hospital, Phitsanulok, Thailand; 3 Department of Orthopedics, Kamphaeng Phet Hospital, Kamphaeng Phet, Thailand; 4 Department of Orthopedics, Naresuan University, Phitsanulok, Thailand

**Keywords:** High tibial osteotomy, Joint line obliquity, Predictive model, Logistic regression, Knee osteoarthritis

## Abstract

**Background:**

The purpose of this study was to identify key preoperative radiographic parameters and develop a predictive model for abnormal knee joint line obliquity (KJLO) following medial open-wedge high tibial osteotomy (MOWHTO) in patients with medial compartment knee osteoarthritis.

**Methods:**

This retrospective observational cohort study included 81 patients (100 knees) treated with MOWHTO. All patients had symptomatic medial knee osteoarthritis with varus alignment and underwent preoperative and postoperative long-standing radiographs under a standardized protocol. Radiographic parameters including preoperative hip-knee angle (HKA), mechanical axis deviation, mechanical lateral distal femoral angle (mLDFA), medial proximal tibial angle (MPTA), joint line congruence angle (JLCA), and KJLO were measured. Multivariable logistic regression analysis was used to identify strong predictors of abnormal postoperative KJLO, defined as an angle > 4°. The final model was selected on the basis of backward elimination method. Model’s discrimination performance was analyzed by using sensitivity, specificity, and area under the receiver operating characteristic curve (AuROC). Model stability was demonstrated by calibration plot and Hosmer–Lemeshow goodness of fit. Bootstrapping internal validation was performed to estimate model accuracy.

**Results:**

Of 81 patients (100 knees), there were 65 female and 16 male patients with an average age of 50.1 years and mean body mass index (BMI) of 27.8 kg/m^2^; 61 knees were found post-KJLO > 4°. According to the performance of logistic regression, sensitivity, specificity, and ROC were calculated to select the best formula for postoperative KJLO prediction. The final predictive model included preoperative HKA, MPTA, and KJLO, and the planned correction angle. The model demonstrated strong performance with an AuROC of 0.876 (95% CI 0.808–0.945). Calibration plot illustrated calibration-in-the-large (CITL) of 0.00, E:O of 1.00, slope of 1.00. Following internal validation, the model remained robust with AuROC of 0.875. The model demonstrated a sensitivity of 80.30%, specificity of 87.20%, positive predictive value of 90.70%, and negative predictive value of 73.90%.

**Conclusions:**

This predictive model incorporating preoperative HKA, MPTA, and KJLO, and the planned correction angle, demonstrates high internal validity and serves as a robust tool for individualized surgical planning in MOWHTO. By identifying high-risk patients preoperatively, surgeons can proactively tailor treatment strategies, such as modifying the target correction angle or opting for other surgical options.

*Trial registration*: TCTR20251003003.

**Supplementary Information:**

The online version contains supplementary material available at 10.1186/s43019-026-00333-5.

## Introduction

Knee osteoarthritis (KOA) is a common degenerative joint disorder, particularly among the elderly, characterized by progressive pain, structural deformity, and functional impairment. Medial open-wedge high tibial osteotomy (MOWHTO) has emerged as a standard joint-preserving surgery for managing unicompartmental medial KOA in active individuals. This procedure functions by realigning the mechanical axis of the lower limb toward the lateral compartment, thereby redistributing load-bearing forces, and potentially delaying the requirement for total knee arthroplasty (TKA) [1, 2].

Despite the established clinical efficacy of MOWHTO, postoperative radiographic assessments frequently demonstrate an increased lateral knee joint line obliquity (KJLO) in the coronal plane [3–5]. This alteration in joint line orientation potentially leads to adverse biomechanical outcomes, including elevated lateral shear stress, articular cartilage degradation, and accelerated progression of osteoarthritis [6, 7]. Specifically, Nakayama et al. [7] reported that a KJLO exceeding 5° doubles the shear stress across the lateral compartment compared with a neutral alignment. For clinical evidence, increased KJLO > 4° was associated with inferior clinical outcomes [4, 8]. Furthermore, variations in tibial plateau inclination and ankle joint orientation have been correlated with increased KJLO, underscoring its broader biomechanical implications [5].

Numerous studies have sought to identify preoperative predictors of postoperative KJLO, focusing on parameters such as baseline joint alignment, femoral and tibial morphology, and the magnitude of surgical correction [4, 9, 10]. Nevertheless, discrepancies persist regarding the most significant predictive factors, and a comprehensive, validated model remains unavailable. While Oh et al. [4] highlighted the importance of the preoperative hip–knee–ankle (HKA) angle and joint line congruence angle (JLCA), Park et al. [10] proposed a logistic regression model on the basis of the lower limb adduction angle, illustrating a lack of consensus on the primary predictive components.

Considering the detrimental impact of abnormal KJLO on long-term clinical outcomes, a robust preoperative predictive model is essential to assist surgeons in identifying high-risk patients and determining optimal interventions, such as double-level osteotomy or TKA [11, 12]. This study aimed to develop a logistic-regression-based model to predict the risk of pathological postoperative KJLO (defined as > 4°) utilizing radiographic parameters. Such a model could serve as a valuable instrument for personalized surgical planning and clinical decision-making for patients undergoing MOWHTO.

## Materials and methods

### Study design and patient selection

This retrospective observational cohort study included patients who underwent MOWHTO at a single institution between January 2015 and December 2022. The study was approved by the Institutional Review Board (IRB) and was registered with trial number: TCTR20251003003.The requirement for informed consent was waived by the IRB due to the retrospective nature of the study.

Eligible participants were those diagnosed with symptomatic medial compartment knee osteoarthritis and varus malalignment. Inclusion criteria required the availability of both preoperative and postoperative orthoroentgenograms (long-standing lower extremity radiographs) obtained using a standardized protocol. Exclusion criteria consisted of valgus deformity, post-traumatic osteoarthritis, concomitant ligament reconstruction, and medial tibial plateau defects. Furthermore, patients with nonstandardized imaging that precluded accurate radiographic measurement were excluded.

### Sample size calculation

**Fig. 1:**
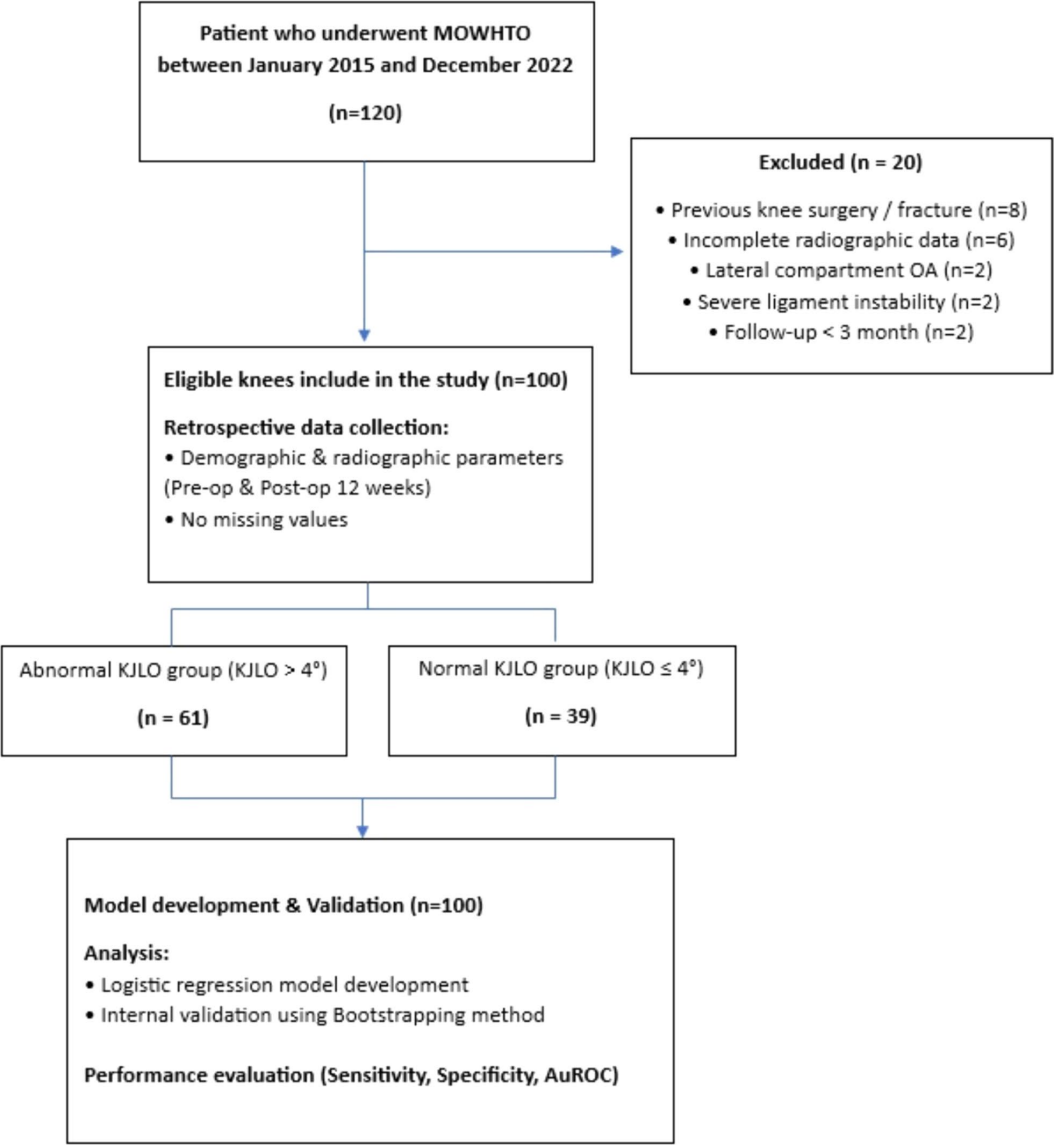
Flow diagram of patient selection

The sample size was calculated on the basis of requirements for multivariable prediction models with binary outcomes, following the guidelines proposed by Peduzzi et al. [13]. Assuming a C-statistic of 0.85, a shrinkage factor of 0.9, and the inclusion of four predictor variables with an estimated prevalence of abnormal KJLO (> 4°) of 0.61 in our clinical setting, a minimum of 87 knees was required. This provided an estimated 13.27 events per variable, with a total of 53 events (postoperative KJLO > 4°) observed in the study population. The flow diagram was shown in Fig. 1.

### Surgical technique and rehabilitation program

All procedures were performed by a single senior orthopedic surgeon using a consistent surgical technique. The surgical approach commenced with an incision over the pes anserinus at the midportion of the anteromedial proximal tibia. The pre-sartorial fascia was identified and incised horizontally along the superior border of the pes anserinus. A small retractor was utilized to protect the pes anserinus tendons (semitendinosus and gracilis), and the anterior third of the superficial medial collateral ligament was released subperiosteally. Under fluoroscopic guidance, a 1.8-mm Kirschner wire was placed from the superior border of the pes anserinus insertion toward the tip of the fibular head to define the osteotomy trajectory. A biplanar V-shaped osteotomy was performed, consisting of a posterior two-thirds cut along the guide wire and an anterior one-third cut angulated at 110° toward the superior border of the tibial tubercle to preserve the extensor mechanism.

The osteotomy was completed using an oscillating saw and chisels, maintaining a 1-cm lateral bone hinge to prevent displacement. A spreader was then utilized to gradually open the osteotomy gap until the mechanical axis passed through the Fujisawa point [14], as confirmed by intraoperative fluoroscopy. Internal fixation was achieved using the TomoFix plate system (Synthes GmbH; Solothurn, Switzerland), with five proximal and two distal locking screws; the latter were inserted percutaneously. No bone graft or substitutes were employed. A Redivac drain was placed, and the wound was closed primarily. Postoperatively, a compressive dressing was applied for 24 h, and sutures were removed at 2 weeks. Early active range of motion and partial weight-bearing with gait assistance were initiated on postoperative day 1, progressing to full weight-bearing by 4–6 weeks.

### Radiographic evaluation

**Fig. 2:**
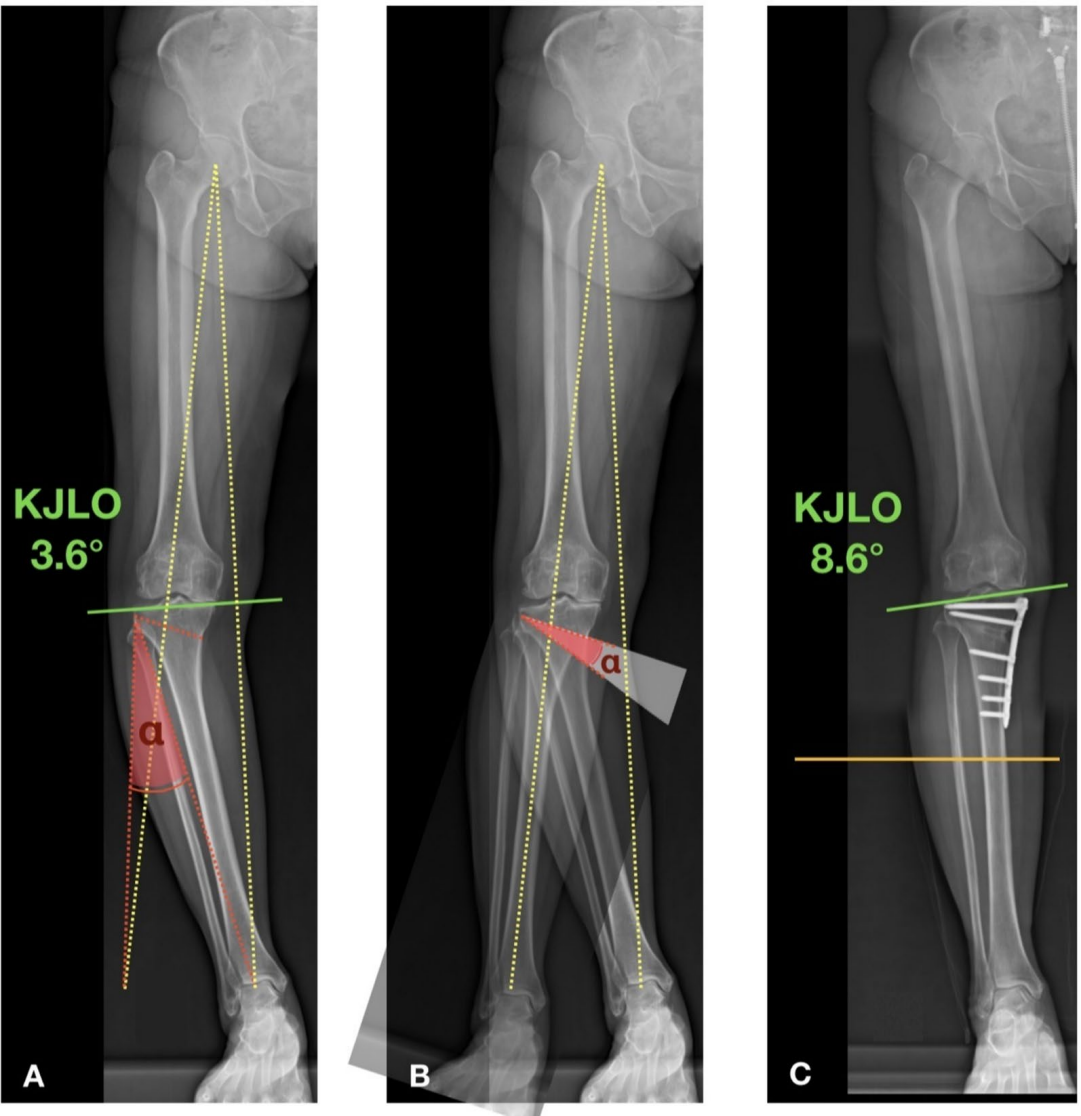
Representative orthoroentgenograms demonstrating preoperative planning and the alteration of lateral knee joint line obliquity (KJLO) following MOWHTO. **A** Preoperative alignment of a patient with a KJLO of 3.6°. **B** Preoperative planning utilizing the Miniaci method [15]; the target correction angle (*α*) is determined by establishing a lateral hinge point to project the new mechanical axis through the Fujisawa point [14]. **C** Postoperative alignment of the same patient showing an increase of KJLO to 8.6°

All preoperative and postoperative orthoroentgenograms (full-length weight-bearing anteroposterior radiographs) were acquired strictly adhering to a standardized institutional protocol established with the Department of Radiology since 2010. Patients were positioned barefoot to eliminate leg-length discrepancies caused by footwear. The inter-foot distance was standardized by placing the feet approximately shoulder-width apart (10–15 cm) to ensure equal weight distribution and prevent anatomical overlap of the knees. To strictly control for lower limb rotation and compensate for femoral anteversion, the feet were positioned in 15 degrees of internal rotation, ensuring both patellae were facing strictly anteriorly (“patella forward” position). A uniform source-to-image distance of 10 feet (305 cm) was maintained, with the central x-ray beam directed at the level of the knee joints. These standardized orthoroentgenograms were utilized for both preoperative planning and postoperative evaluation. The target correction angle was calculated preoperatively utilizing the Miniaci method [15], aiming to shift the mechanical weight-bearing axis to the Fujisawa point (the lateral 62.5% of the tibial plateau) [14] (Fig. 2). Six preoperative parameters were assessed as potential predictors of postoperative KJLO: (1) hip–knee–ankle (HKA) angle, (2) mechanical lateral distal femoral angle (mLDFA), (3) medial proximal tibial angle (MPTA), (4) joint line congruence angle (JLCA), (5) preoperative KJLO, and (6) correction angle.

**Fig. 3:**
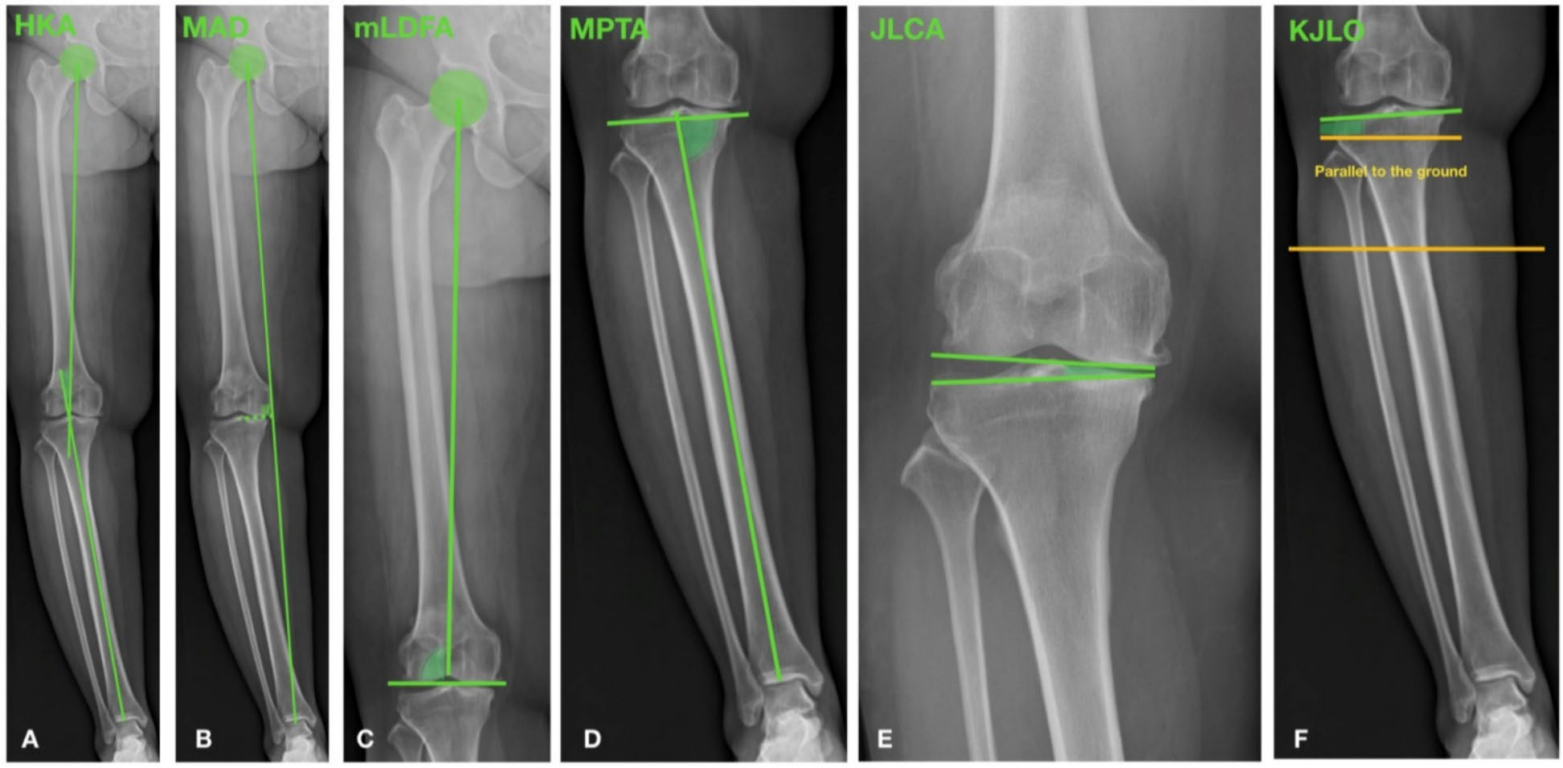
Illustration of preoperative radiographic parameters: **A** hip–knee–ankle (HKA) angle; **B** mechanical axis deviation (MAD); **C** mechanical lateral distal femoral angle (mLDFA); **D** medial proximal tibial angle (MPTA); **E** joint line congruence angle (JLCA); and **F** knee joint line obliquity (KJLO)

The HKA angle was defined as the angle between the mechanical femoral axis (a line from the center of the femoral head to the intercondylar notch) and the mechanical tibial axis (a line from the center of the tibial plateau to the center of the ankle joint), with negative values indicating valgus alignment (Fig. 3A). Mechanical axis deviation (MAD) was measured as the distance between the mechanical axis and the center of the tibial plateau; negative values represented an axis passing lateral to the tibial midline (Fig. 3B). The mLDFA was the lateral angle between the mechanical femoral axis and the distal femoral joint line (Fig. 3C). The MPTA was the medial angle between the mechanical tibial axis and the proximal tibial joint line (Fig. 3D). The JLCA was the angle between the line tangent to the distal femoral condyles and the tibial plateau, with negative values indicating lateral joint space opening (Fig. 3E). The KJLO was defined as the angle between a line parallel to the floor and the joint line of the tibial condyles, with negative values representing lateral inclination (Fig. 3F). Finally, the difference between preoperative and postoperative MPTA represented the actual magnitude of the preoperative planned correction angle.

### Data collection and analysis

A total of 81 patients, 62 unilateral knee and 19 staged bilateral MOWHTO (100 knees) meeting the inclusion criteria were enrolled in this study (Fig. 1). Demographic and clinical data, including age, sex, BMI, surgical side, and the severity of knee osteoarthritis (KOA), were retrieved from the institutional electronic medical record system. Radiographic parameters—including HKA, mLDFA, MPTA, KJLO, and correction angle—were measured using the Picture Archiving and Communication System (PACS) at both preoperative and 12-week postoperative intervals. To ensure data reliability, all radiographic measurements were performed by an independent orthopedic surgeon blinded to the clinical data; each parameter was measured twice to ensure consistency, and no missing data were identified within the final dataset.

In this study, a postoperative KJLO > 4° was defined as significant lateral joint line obliquity, because recent clinical studies demonstrated that inferior postoperative clinical outcomes begin to manifest at a threshold of > 4° [4, 8, 16]. Baseline characteristics were compared using Fisher’s exact test for categorical variables and independent *t*-tests for continuous variables. Univariable and multivariable logistic regression analyses were conducted to identify preoperative predictors of postoperative KJLO > 4°. Both crude and adjusted odds ratios (ORs) with 95% confidence intervals (CIs) were reported.

The final predictive model was developed using a backward elimination approach with a removal criterion of *p* > 0.05, however, clinical relevance was considered during model selection. For instance, preoperative KJLO was retained in the final model despite lacking independent statistical significance, given its importance as a baseline anatomical parameter and its contribution to maximizing the model’s discriminative ability. Model performance was evaluated using the area under the receiver operating characteristic curve (AuROC) for discrimination. Model calibration was assessed via calibration plots, the slope of the calibration line, the expected-to-observed (E:O) ratio, and calibration-in-the-large (CITL). To evaluate internal validity and prevent overfitting, bootstrapping with 1000 resamples was performed. Furthermore, the clinical utility of the model was appraised using decision curve analysis (DCA), comparing it against “treat-all” and “treat-none” strategies. Finally, the discriminative ability of the final model was further characterized by calculating sensitivity, specificity, positive predictive value (PPV), negative predictive value (NPV), and positive and negative likelihood ratios (LHR+ and LHR−).

## Results

Of 100 knees (81 patients), 81 (81.0%) were from female patients, with an average age of 50.09 ± 5.97 years and mean BMI of 27.84 ± 4.02 kg/m^2^. Means of preoperative and postoperative MAD, HKA, mLDFA, JLCA, and planned correction angle compared between groups were demonstrated as statistically significant (Table 1), and 61 (61.0%) knees were found abnormal postoperative KJLO > 4°.

**Table 1:** Baseline demographic and radiographic characteristics of patients, stratified by postoperative knee joint line obliquity (KJLO > 4° versus ≤ 4°)

Prognostic factor	Postoperative KJLO > 4° (mean ± SD)	Postoperative KJLO ≤ 4° (mean ± SD)	*p*-Value
Age (years)	50.16 ± 0.67	49.97 ± 1.13	0.878
Female, *n* (%)	50 (81.97)	31 (79.49)	0.798
BMI (kg/m^2^)	28.64 ± 0.51	26.60 ± 0.61	0.013*
Right knee, *n* (%)	30 (49.18)	18 (46.15)	0.839
Diagnosis, *n* (%)			0.901
Medial compartment OA	34 (55.74)	21 (53.85)	
Medial and PF compartment OA	26 (42.46)	18 (46.15)	
Medial compartment OA with ACL torn	1 (1.64)	0 (0.00)	
Tibial width (cm)	76.16 ± 0.64	77.59 ± 0.99	0.531
Mechanical axis deviation (mm)	42.48 ± 1.62	30.92 ± 2.45	< 0.001*
HKA (°)	11.85 ± 0.46	8.58 ± 0.76	< 0.001*
mLDFA (°)	89.72 ± 0.24	88.21 ± 0.37	< 0.001*
MPTA (°)	84.36 ± 0.34	83.93 ± 0.63	0.306
JLCA (°)	6.48 ± 0.34	4.17 ± 0.43	< 0.001*
KJLO (°)	2.60 ± 0.28	1.63 ± 0.46	0.063
Posterior tibial slope (°)	6.69 ± 0.48	6.22 ± 0.80	0.593
Planned correction angle (°)	12.26 ± 0.47	9.90 ± 0.57	0.002*

*KOA* knee osteoarthritis, *BMI* body mass index, *HKA* hip–knee–ankle angle, *mLDFA* mechanical lateral distal femoral angle, *MPTA* medial proximal tibial angle, *JLCA* joint line congruence angle, *KJLO* knee joint line obliquity, *kg/m*^*2*^ kilograms/meters^2^, *cm* centimeters, *°* degree

^*^Statistical significance

**Fig. 4:**
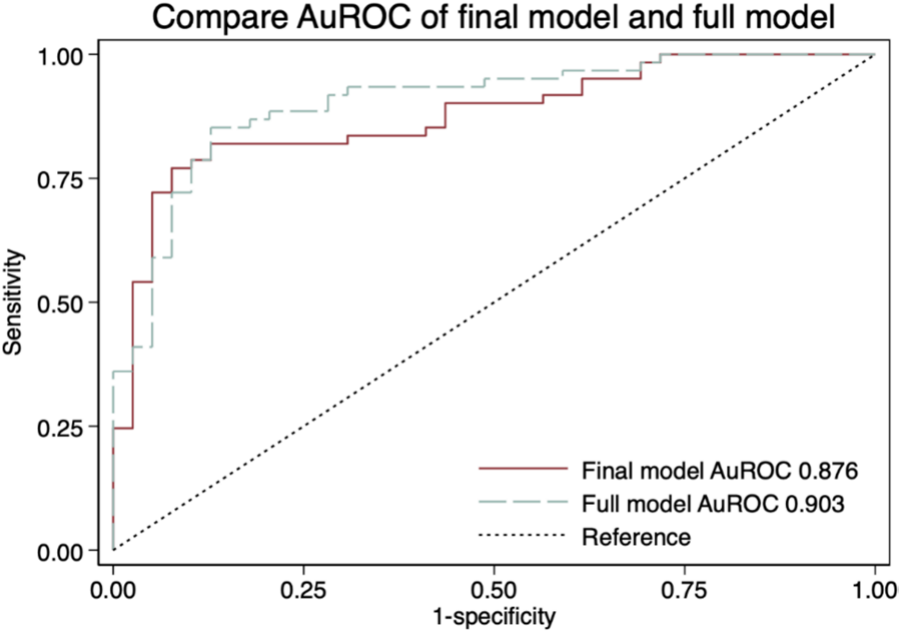
Comparison of the area under the receiver operating characteristic (AuROC) curves between the full multivariable model and the final parsimonious model for predicting abnormal postoperative KJLO

Multivariable logistic regression analysis was performed to develop the predictive model. The full model, incorporating all initial parameters, demonstrated an AuROC of 0.903 (95% CI 0.842–0.964) (Table 2). Following a backward elimination process, the final model identified four significant predictors: preoperative HKA (aOR: 1.57; 95% CI 1.24–1.97; *p* < 0.001), preoperative MPTA (aOR: 2.00; 95% CI 1.14–2.83; *p* < 0.001), preoperative KJLO (aOR: 0.88; 95% CI 0.67–1.15; *p* = 0.349), and the planned correction angle (aOR: 1.43; 95% CI 1.18–1.75; *p* < 0.001) (Table 3). The final model exhibited strong discriminative performance with an AuROC of 0.876 (95% CI 0.808–0.945) (Fig. 4). The predictive equation was derived as follows:

$${\text{Predicted Probability}}\, = \,\frac{{e^{{\left( { - 66.23233\, + \,0.4475727*x1\, + \,0.6936062*x2\, - \,0.1291533*x3\, + \,0.3593394*x4} \right)}} }}{{1\, + \,e^{{\left( { - 66.23233\, + \,0.4475727*x1\, + \,0.6936062*x2\, - \,0.1291533*x3\, + \,0.3593394*x4} \right)}} }}$$

**Table 2:** Univariable and multivariable logistic regression analyses of preoperative predictors for significant postoperative knee joint line obliquity (KJLO > 4°)

Prognostic factor	Crude odds ratio	AuROC	*p*-Value	Adjusted odds ratio^**^	*p*-Value
	Odds ratio (95% CI)			Odds ratio (95% CI)	
Age (years)	1.01 (0.94, 1.07)	0.4968	0.876	1.06 (0.93, 1.20)	0.234
Female	1.17 (0.43, 3.24)	0.5124	0.758	0.20 (0.01, 2.83)	0.390
BMI (kg/m^2^)	1.15 (1.03, 1.28)	0.6295	0.016*	1.16 (0.97, 1.39)	0.106
Right	1.13 (0.50, 2.53)	0.5151	0.768	1.58 (0.49, 5.08)	0.442
Diagnosis	0.99 (0.46, 2.15)	0.5057	0.981	0.45 (0.13, 1.53)	0.200
Tibial width (cm)	0.98 (0.91, 1.05)	0.5216	0.527	0.95 (0.79, 1.13)	0.565
Mechanical axis deviation (mm)	1.06 (1.03, 1.10)	0.7575	< 0.001*	1.02 (0.77, 1.34)	0.893
HKA (°)	1.23 (1.10, 1.38)	0.7562	0.001*	1.21 (0.42, 3.47)	0.729
mLDFA (°)	1.42 (1.14, 1.76)	0.6938	0.002*	1.26 (0.66, 2.43)	0.483
MPTA (°)	1.07 (0.94, 1.20)	0.5105	0.314	1.82 (1.02, 3.25)	0.043*
JLCA (°)	1.42 (1.18, 1.71)	0.7421	< 0.001*	1.37 (0.74, 2.54)	0.320
KJLO (°)	1.17 (0.99, 1.39)	0.6326	0.068	0.83 (0.60, 1.16)	0.283
Posterior tibial slope (°)	1.03 (0.93, 1.12)	0.5240	0.589	0.94 (0.80, 1.10)	0.456
Planned correction angle (°)	1.20 (1.06, 1.35)	0.7396	0.004*	1.59 (1.23, 2.04)	< 0.001*

*AuROC* area under receiver operating characteristic curve, *CI* confidence interval, *BMI* body mass index, *HKA* hip–knee–ankle angle, *mLDFA* mechanical lateral distal femoral angle, *MPTA* medial proximal tibial angle, *JLCA* joint line congruence angle, *KJLO* knee joint line obliquity, *kg/m*^*2*^ kilograms/meters^2^, *cm* centimeters, *°* degree

*p* < 0.05 were considered statistically significant

^*^Statistical significance

^**^Adjusted AuROC 0.903; 95% CI 0.842–0.964

**Table 3:** Final multivariable logistic regression model for predicting significant postoperative knee joint line obliquity (KJLO > 4°) after backward elimination

Prognostic factor	*ß*	SE	Adjusted odds ratio	*p*-Value
			Odds ratio (95% CI)	
HKA (°)	0.45	0.12	1.57 (1.24, 1.97)	< 0.001*
MPTA (°)	0.69	0.18	2.00 (1.14, 2.83)	< 0.001*
KJLO (°)	−0.13	0.14	0.88 (0.67, 1.15)	0.349
Planned correction angle (°)	0.36	0.10	1.43 (1.18, 1.75)	< 0.001*

*CI* confidence interval, *HKA* hip–knee–ankle angle, *MPTA* medial proximal tibial angle, *KJLO* knee joint line obliquity

^*^Statistical significance

**Fig. 5:**
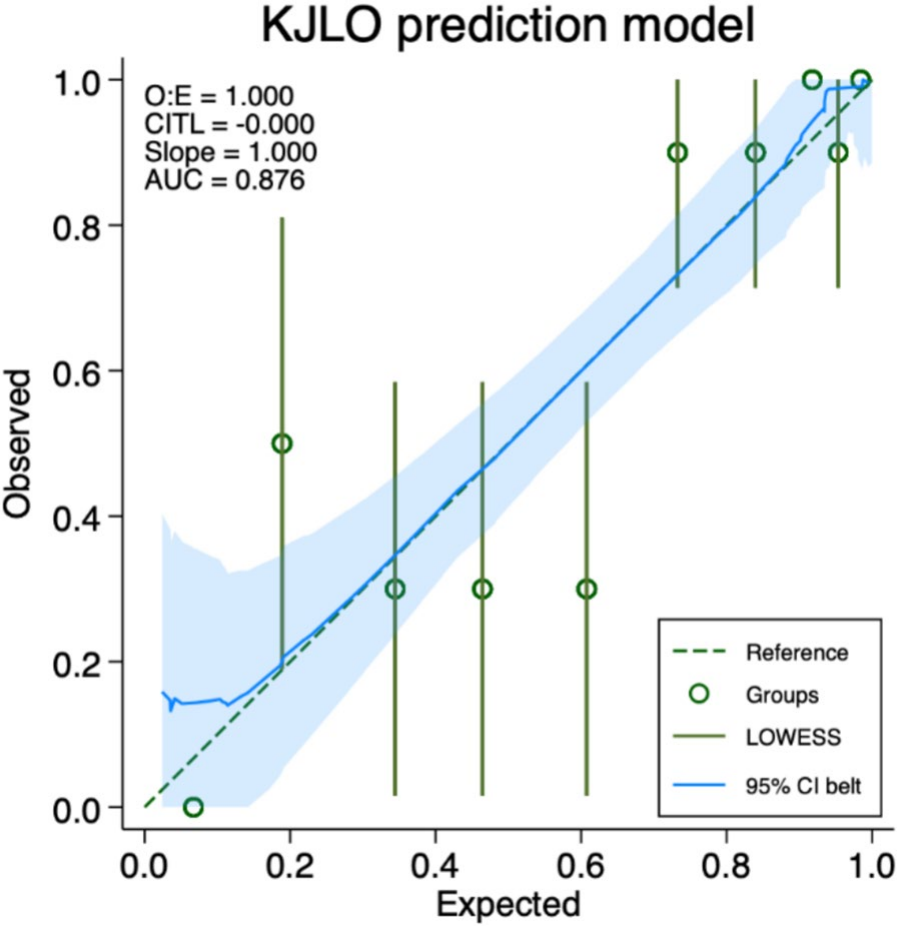
Calibration plot of the final predictive model, illustrating the relationship between the predicted probability and the observed frequency of abnormal postoperative KJLO (> 4°)

**Fig. 6:**
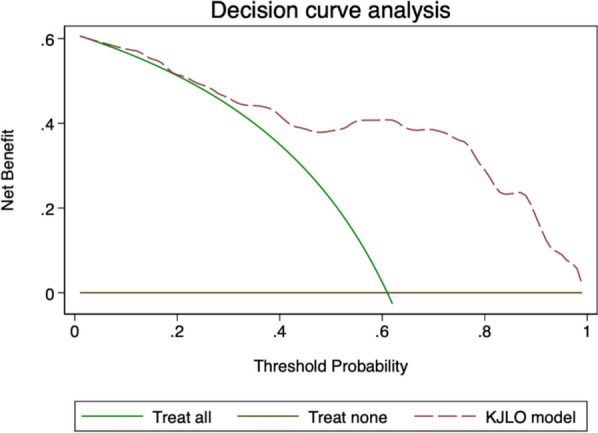
Decision curve analysis demonstrating the clinical net benefit of the final predictive model compared to the “treat-all” (intervention for all patients) and “treat-none” (no intervention) strategies across various threshold probabilities

Model calibration assessment demonstrated a slope of 1.000 (95% CI 0.604–1.396), a calibration-in-the-large (CITL) of 0.000 (95% CI −0.527 to 0.527), and an E:O ratio of 1.000. Following internal validation via bootstrapping (1,000 resamples), the model remained robust with a calibration slope of 0.984 (95% CI 0.598–1.434), a CITL of 0.014 (95% CI −0.548 to 0.636), and an E:O ratio of 0.998. The AuROC after bootstrapping was 0.875 (95% CI 0.812–0.954) (Table 4 and Fig. 5). Furthermore, decision curve analysis (DCA) indicated that the clinical utility of the predictive model provided a higher net benefit compared with both “treat-all” and “treat-none” strategies across the relevant range of threshold probabilities (Fig. 6).

**Table 4:** Internal validation of the predictive model using bootstrapping with 1000 resamples

Statistic	Model performance	
	Apparent	Bootstrapping
AuROC (95% CI)	0.876 (0.808, 0.945)	0.875 (0.812, 0.954)
E:O ratio (95% CI)	1.000	0.998
CITL (95% CI)	0.000 (−0.527, 0.527)	0.014 (−0.548, 0.636)
Slope (95% CI)	1.000 (0.604, 1.396)	0.984 (0.598, 1.434)
Bootstrap shrinkage = 0.984		

*AuROC* area under the receiver operating characteristic curve, *E:O ratio* expected-to-observed ratio, *CITL* calibration-in-the-large, *CI* confidence interval

**Fig. 7:**
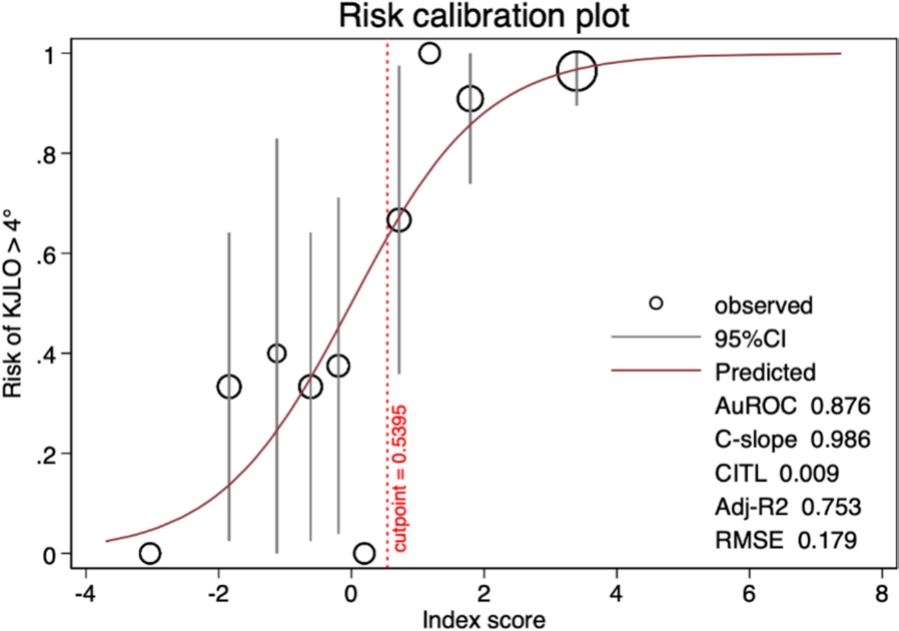
Risk calibration plot comparing the observed versus predicted risks of abnormal postoperative KJLO (> 4°), evaluated at the selected diagnostic threshold

To determine the optimal cut-point, a threshold was established on the basis of the 61.0% prevalence of abnormal KJLO (> 4°) in our study, resulting in an index score of 0.5395. Using this threshold, 54 knees (54.0%) were classified as high risk, with a false positive rate of 5.0% (5 knees). The model demonstrated a sensitivity of 80.30% (95% CI 68.20–89.40%), a specificity of 87.20% (95% CI 72.60–95.70%), a positive predictive value of 90.70% (95% CI 79.70–96.90%), and a negative predictive value of 73.90% (95% CI 58.90–85.70%) (Table 5). Furthermore, a risk calibration plot (Fig. 7) confirmed the strong correlation between the observed and predicted risks of abnormal KJLO at the selected cut-point.

**Table 5:** Confusion matrix and diagnostic performance metrics of the predictive model for abnormal postoperative KJLO (> 4°), stratified by risk level

Predicted	KJLO > 4°	KJLO ≤ 4°	Total
	*n* (%)	*n* (%)	*n* (%)
High-risk KJLO > 4°	49 (49)	5 (5)	54 (54)
Low-risk KJLO > 4°	12 (12)	34 (34)	46 (46)
Total	61 (61)	39 (39)	100 (100)
Sensitivity 80.30 (95% CI 68.20–89.40)			
Specificity 87.20 (95% CI 72.60–95.70)			
Positive predictive value 90.70 (95% CI 79.70–96.90)			
Negative predictive value 73.90 (95% CI 58.90–85.70)			
Likelihood ratio (+) 6.27 (95% CI 2.74–14.34)			
Likelihood ratio (−) 0.23 (95% CI 0.13–0.38)			

*KJLO* knee joint line obliquity, *CI* confidence interval

## Discussion

Lateral KJLO is a frequently encountered radiographic phenomenon following MOWHTO. While the primary objective of MOWHTO is to shift the mechanical axis toward the lateral compartment to unload the degenerative medial compartment, excessive correction may inadvertently lead to abnormal joint line orientation. The clinical implications of such alterations are significant. Nakayama et al. [7] demonstrated that a postoperative KJLO exceeding 5° resulted in a twofold increase in shear stress on the lateral articular cartilage compared with a neutral orientation. Furthermore, Kim et al. [6] reported that patients with increased lateral KJLO experienced higher rates of lateral compartment pain at a minimum 4-year follow-up. Although the acceptable range of postoperative KJLO is between 4° and 5° [3, 6, 7], recent midterm clinical studies indicated that inferior clinical outcomes begin to appear at > 4° [4, 8, 16]. Consequently, the prevention of abnormal KJLO > 4° may minimize the risk of accelerated cartilage degeneration and ensure optimal long-term clinical outcomes.

Despite its clinical significance, predicting which patients are at risk for developing abnormal KJLO remains challenging. Previous predictive models have yielded inconsistent results regarding the primary determinants [4, 10, 17]. Our study addresses this gap by developing a robust logistic regression-based model using readily available preoperative radiographic parameters. The final model incorporates four variables—preoperative HKA, MPTA, and KJLO, and the planned correction angle—demonstrating high predictive performance with an AuROC of 0.876, sensitivity of 80.3%, and specificity of 87.2%. The ability to predict abnormal KJLO preoperatively provides substantial clinical value. For patients identified as “high risk” by our model, surgeons can consider patient-specific modifications to the surgical plan. These strategies may include reducing the intended correction angle to accept a mild varus alignment, or in cases requiring substantial correction, performing a double-level osteotomy (DLO) to preserve joint line orientation [11, 12]. Consequently, this model empowers individualized decision-making and facilitates informed preoperative counseling between surgeons and patients.

Our findings offer a refined perspective compared with previous literature. While Oh et al. [4] emphasized the significance of the preoperative joint line congruence angle (JLCA), our multivariate analysis identified structural parameters, specifically HKA and MPTA, as more dominant predictors. Similarly, whereas Park et al. [10] utilized the lower limb adduction angle, our model employs standard radiographic indices that are routinely measured in daily practice, thereby enhancing its clinical utility and ease of implementation. A distinct strength of our study is the integration of the “planned correction angle” as a key variable. Unlike previous models that rely solely on native anatomy, our approach accounts for the magnitude of surgical correction—the only modifiable factor during preoperative planning. The inclusion of this variable allows surgeons to dynamically assess how various degrees of correction might alter the risk of abnormal KJLO for a specific patient, transforming the model from a purely prognostic tool into a practical surgical planning aid.

Crucially, our data highlight a significant dissociation between mechanical alignment and joint line orientation. Achieving a neutral mechanical axis does not inherently guarantee a physiological joint line. We observed multiple instances where the mechanical axis was successfully corrected to the target Fujisawa point, yet the patients still exhibited abnormal postoperative KJLO (> 4°). Previous study demonstrated preoperative KJLO, correction angle, preoperative HKA, and preoperative MPTA were strongly factor of postoperative KJLO [4, 10], and therefore we have selected the factors on the basis of these clinical relevances. Although our multivariable logistic regression including all prognostic factors showed that KJLO was not a significant predictor, we found that final model that included preoperative MPTA, planed correction angle, preoperative HKA, and preoperative KJLO had the highest AuROC after performing sensitivity analysis. We hypothesized that the effect of KJLO might be masked by primary structural deformities (MPTA) and the magnitude of surgical intervention. We observed that the severe varus deformities, particularly in patients with a high BMI, the medial soft tissues are exceptionally tight. Achieving the massive target correction frequently requires a progressive release extending further posteriorly—incorporating up to two-thirds of the superficial medial collateral ligament (MCL), the deep MCL, and the medial periosteum. This extensive release inevitably destabilizes the proximal tibial fragment. Consequently, the postoperative joint line orientation becomes highly unpredictable and is dictated almost entirely by the magnitude of this soft tissue release and the planned correction angle [18, 19]. These findings underscores the necessity of evaluating coronal joint line geometry as a distinct and independent surgical goal, rather than assuming it will normalize solely through correction of the mechanical axis.

Despite these strengths, several limitations of this study should be acknowledged. First, the predictive model was developed from a cohort of patients with varus-aligned medial compartment knee osteoarthritis; therefore, its applicability to patients with valgus deformities or those undergoing varus-producing distal femoral osteotomy remains to be established. Second, although we utilized the preoperative joint line congruence angle (JLCA) as a validated radiographic surrogate to account for coronal soft tissue laxity, we did not perform specific quantitative clinical laxity tests or utilize stress radiographs. While preoperative JLCA showed a significant difference in the univariable analysis, it did not remain a strong independent predictor in the final multivariable model, suggesting that bony structural deformities had a more dominant influence in our cohort. However, the lack of direct, dynamic soft tissue tension measurements limits our ability to fully and precisely evaluate the distinct contribution of ligamentous influence on postoperative KJLO, which should be addressed in future prospective studies. Third, this study included 19 patients with staged bilateral MOWHTO (38 knees). While we analyzed these cases as independent observations to maintain a straightforward, coefficient-based predictive scoring model for clinical practice, additional sensitivity analysis using a multilevel logistic regression model was performed to evaluate the potential clustering effect introduced by the staged bilateral cases. The multilevel model demonstrated an overall discriminative performance nearly identical to the primary model, with an AuROC of 0.874 (95% CI 0.805–0.943). Internal validation via bootstrapping confirmed model stability (bootstrapped AuROC: 0.873, 95% CI 0.810–0.945; calibration slope: 0.987). Nevertheless, due to the limited proportion of clustered data (19%), the multilevel model exhibited inflated standard errors and wider 95% confidence intervals as detailed in the supplementary material. Finally, although internal validation using bootstrapping demonstrated high model stability and performance, external validation in independent, multicenter cohorts is warranted to confirm the generalizability of our findings across diverse populations.

## Conclusions

Our predictive model incorporating preoperative HKA, MPTA, KJLO, and the planned correction angle demonstrates high internal validity and serves as a robust tool for individualized surgical planning in MOWHTO. By identifying high-risk patients preoperatively, surgeons can proactively tailor treatment strategies, such as modifying the target correction angle or opting for a double-level osteotomy. Implementation of this model may optimize postoperative biomechanical outcomes, minimize abnormal joint line orientation, and ultimately enhance long-term patient satisfaction.

## Supplementary Information

Supplementary material 1.

## Data Availability

The datasets used and/or analyzed during the current study are available from the corresponding author on reasonable request.

## References

[1] Bode G, von Heyden J, Pestka J, Schmal H, Salzmann G, Südkamp N, Niemeyer P (2015) Prospective 5-year survival rate data following open-wedge valgus high tibial osteotomy. Knee Surg Sports Traumatol Arthrosc 23(7):1949–1955 24241123 10.1007/s00167-013-2762-y

[2] Floerkemeier S, Staubli AE, Schroeter S, Goldhahn S, Lobenhoffer P (2013) Outcome after high tibial open-wedge osteotomy: a retrospective evaluation of 533 patients. Knee Surg Sports Traumatol Arthrosc 21(1):170–180 22744433 10.1007/s00167-012-2087-2

[3] Tseng TH, Wang HY, Tzeng SC, Hsu KH, Wang JH (2022) Knee-ankle joint line angle: a significant contributor to high-degree knee joint line obliquity in medial opening wedge high tibial osteotomy. J Orthop Surg Res 17(1):79 35123546 10.1186/s13018-022-02976-y PMC8818150

[4] Oh KJ, Ko YB, Bae JH, Yoon ST, Kim JG (2016) Analysis of knee joint line obliquity after high tibial osteotomy. J Knee Surg 29(8):649–657 26838969 10.1055/s-0036-1571430

[5] Lee KM, Chang CB, Park MS, Kang SB, Kim TK, Chung CY (2015) Changes of knee joint and ankle joint orientations after high tibial osteotomy. Osteoarthr Cartil 23(2):232–238 10.1016/j.joca.2014.11.001 25450843

[6] Kim GW, Kang JK, Song EK, Seon JK (2021) Increased joint obliquity after open-wedge high tibial osteotomy induces pain in the lateral compartment: a comparative analysis of the minimum 4-year follow-up outcomes using propensity score matching. Knee Surg Sports Traumatol Arthrosc 29(10):3495–3502 33151363 10.1007/s00167-020-06342-5

[7] Nakayama H, Schröter S, Yamamoto C, Iseki T, Kanto R, Kurosaka K, Kambara S, Yoshiya S, Higa M (2018) Large correction in opening wedge high tibial osteotomy with resultant joint-line obliquity induces excessive shear stress on the articular cartilage. Knee Surg Sports Traumatol Arthrosc 26(6):1873–1878 28831525 10.1007/s00167-017-4680-x

[8] Song JH, Bin SI, Kim JM, Lee BS (2020) What is an acceptable limit of joint-line obliquity after medial open wedge high tibial osteotomy? Analysis based on midterm results. Am J Sports Med 48(12):3028–3035. 10.1177/0363546520949552 32941061 10.1177/0363546520949552

[9] Victor JM, Bassens D, Bellemans J, Gürsu S, Dhollander AA, Verdonk PC (2014) Constitutional varus does not affect joint line orientation in the coronal plane. Clin Orthop Relat Res 472(1):98–104 23733590 10.1007/s11999-013-2898-6 PMC3889437

[10] Park JG, Bin SI, Kim JM, Lee BS (2021) Using the lower limb adduction angle to predict postoperative knee joint-line obliquity after open-wedge high tibial osteotomy. Orthop J Sports Med 9(5):23259671211003990 34026916 10.1177/23259671211003991 PMC8120547

[11] Akamatsu Y, Nejima S, Tsuji M, Kobayashi H, Muramatsu S (2022) Joint line obliquity was maintained after double-level osteotomy, but was increased after open-wedge high tibial osteotomy. Knee Surg Sports Traumatol Arthrosc 30(2):688–697 33433634 10.1007/s00167-020-06430-6

[12] Babis GC, An KN, Chao EY, Rand JA, Sim FH (2002) Double level osteotomy of the knee: a method to retain joint-line obliquity. J Bone Joint Surg Am 84(8):1380–1388 12177268 10.2106/00004623-200208000-00013

[13] Peduzzi P, Concato J, Kemper E, Holford TR, Feinstein AR (1996) A simulation study of the number of events per variable in logistic regression analysis. J Clin Epidemiol 49(12):1373–1379 8970487 10.1016/s0895-4356(96)00236-3

[14] Fujisawa Y, Masuhara K, Shiomi S (1979) The effect of high tibial osteotomy on osteoarthritis of the knee. An arthroscopic study of 54 knee joints. Orthop Clin North Am 10(3):585–608 460834

[15] Miniaci A, Ballmer FT, Ballmer PM, Jakob RP (1989) Proximal tibial osteotomy. A new fixation device. Clin Orthop Relat Res 246:250–9 2766613

[16] Hiramatsu K, Yamada Y, Nakamura N, Mitsuoka T (2022) Factors associated with postoperative knee joint line obliquity after medial open wedge high tibial osteotomy. Am J Sports Med 50(6):1651–1658 35293800 10.1177/03635465221079343

[17] Park JY, Chang CB, Kang DW, Oh S, Kang SB, Lee MC (2019) Development and validation of a prediction model for knee joint line orientation after high tibial osteotomy. BMC Musculoskelet Disord 20(1):434 31526379 10.1186/s12891-019-2820-9 PMC6747748

[18] Seitz AM, Nelitz M, Ignatius A, Dürselen L (2019) Release of the medial collateral ligament is mandatory in medial open-wedge high tibial osteotomy. Knee Surg Sports Traumatol Arthrosc 27(9):2917–2926 30269168 10.1007/s00167-018-5167-0

[19] Bagherifard A, Jabalameli M, Mirzaei A, Khodabandeh A, Abedi M, Yahyazadeh H (2020) Retaining the medial collateral ligament in high tibial medial open-wedge osteotomy mostly results in post-operative intra-articular gap reduction. Knee Surg Sports Traumatol Arthrosc 28(5):1388–1393 30972467 10.1007/s00167-019-05473-8

