# Supplementary material for: Prediction model for abnormal joint line obliquity after open wedge high tibial osteotomy using knee radiographic parameters
Source: Knee Surg Relat Res. 2026 Aug 4;38:37. doi: 10.1186/s43019-026-00333-5 (PMC13435420; doi:10.1186/s43019-026-00333-5)
Supplement: Supplementary file 1 [file 43019_2026_333_MOESM1_ESM.docx]

**Supplement material**

Supplemental sensitivity analysis using a multilevel logistic regression model (random-intercept model) was performed to account for the clustering effect of the 19 staged bilateral cases (38 knees). The multilevel analysis demonstrated that the overall predictive performance and stability of our model remain highly consistent. The multilevel model yielded an area under the receiver operating characteristic curve (AuROC) of 0.873 (95% CI: 0.805-0.943) **(Table S1, Table S2, Figure S1)**, which is nearly identical to the primary model's AuROC of 0.876. Internal validation via bootstrapping further confirmed this stability, showing an optimism-corrected AuROC of 0.873 (95% CI: 0.810–0.945) and a calibration slope of 0.987 **(Table S3)**.

We noted that individual predictors lost independent statistical significance in the final multilevel models, accompanied by inflated standard errors and wider 95% confidence intervals (e.g., final model MPTA adjusted OR: 4.34, 95% CI: 0.31–61.38) **(Table S2)**. This is an expected statistical consequence when applying cluster-adjusted models to a dataset with a limited proportion of clustered observations (only 19% bilateral cases). This sparse cluster structure inflates standard errors and limits the power to detect significance for individual variables, even though the overall discriminative capability (AuROC) and calibration remain highly stable.

These findings may imply that intra-patient correlation does not compromise the overall predictive validity of the model, while justifying our use of the primary standard logistic regression to derive a practical, stable coefficient-based scoring system for clinical use.

**Table S1.** Preoperative predictors of post-operative KJLO >4° using multilevel logistic regression analysis.

| Prognostic factor | Adjusted odds ratio^**^ | p-value |
| --- | --- | --- |
|  | odds ratio (95%CI) |  |
| Age (years) | 1.06 (0.93,1.20) | 0.240 |
| female | 0.20 (0.01,2.20) | 0.371 |
| BMI (kg/m^2^) | 1.16 (0.97,1.38) | 0.107 |
| Right | 1.56 (0.49,5.02) | 0.454 |
| diagnosis | 0.46 (0.14,1.56) | 0.215 |
| Tibial width (cm) | 0.94 (0.79,1.13) | 0.504 |
| Mechanical axis deviation (mm) | 1.06 (0.92,1.23) | 0.415 |
| HKAA (°) | 1.21 (0.42,3.47) | 0.729 |
| mLDFA (°) | 1.32 (0.73,2.39) | 0.483 |
| MPTA (°) | 1.74 (1.04,2.91) | 0.033* |
| JLCA (°) | 1.41 (0.78,2.54) | 0.253 |
| KJLO (°) | 0.83 (0.61,1.17) | 0.311 |
| Posterior tibial slope (°) | 0.94 (0.80,1.09) | 0.408 |
| Planned correction angle (°) | 1.58 (1.23,2.02) | <0.001* |

Abbreviations: CI, confidence interval; BMI, body mass index; HKA, hip-knee-ankle angle; mLDFA, mechanical lateral distal femoral angle; MPTA, medial proximal tibial angle; JLCA, joint line congruence angle; KJLO, knee joint line obliquity; kg/m^2^, kilograms/meters^2^; cm, centimeters; °, degree

* Statistical significance, **Adjusted AuROC = 0.903

**Table S2.** Final multilevel regression model for predicting significant postoperative knee joint line obliquity (KJLO >4°)

| Prognostic factor | ß | SE | Adjusted Odds ratio* | p value |
| --- | --- | --- | --- | --- |
|  |  |  | odds ratio (95%CI) |  |
| HKAA (°) | 0.88 | 0.80 | 2.41 (0.50,11.61) | 0.270 |
| MPTA (°) | 1.47 | 1.35 | 4.34 (0.31,61.38) | 0.277 |
| KJLO (°) | -0.30 | 0.38 | 0.74 (0.35,1.57) | 0.429 |
| Planned correction angle (°) | 0.81 | 0.85 | 2.27 (0.42,12.19) | 0.339 |

Abbreviations: CI, confidence interval; HKA, hip-knee-ankle angle; MPTA, medial proximal tibial angle; KJLO, knee joint line obliquity

*AuROC = 0.873 (95% CI: 0.805-0.943)

**Table S3.** Internal validation of the multilevel model using the bootstrapping method.

| Statistic | Model performance | | |
| --- | --- | --- | --- |
|  | Apparent |  | Bootstrapping |
| AuROC (95%CI) | 0.874 (0.805,0.943) |  | 0.873 (0.810,0.945) |
| E:O ratio (95%CI) | 1.000 |  | 0.999 |
| CITL (95%CI) | 0.000 (-0.527,0.527) |  | 0.015 (-0.596,0.639) |
| Slope (95%CI) | 1.000 (0.607,1.393) |  | 0.987 (0.636,1.405) |
| Bootstrap shrinkage = 0.979 | | | |

Abbreviations: AuROC, area under the receiver operating characteristic curve; E:O ratio, expected-to-observed ratio; CITL, calibration-in-the-large; CI, confidence interval

**Figure S1.** Calibration plot of multilevel model, illustrating the relationship between the predicted probability and the observed frequency of abnormal postoperative KJLO (>4°).
